# The need to accessorize: molecular roles of HTLV-1 p30 and HTLV-2 p28 accessory proteins in the viral life cycle

**DOI:** 10.3389/fmicb.2013.00275

**Published:** 2013-09-17

**Authors:** Rajaneesh Anupam, Rami Doueiri, Patrick L. Green

**Affiliations:** ^1^Center for Retrovirus Research, The Ohio State UniversityColumbus, OH, USA; ^2^Department of Veterinary Biosciences, The Ohio State UniversityColumbus, OH, USA; ^3^Comprehensive Cancer Center and Solove Research Institute, The Ohio State UniversityColumbus, OH, USA; ^4^Department of Molecular Virology, Immunology, and Medical Genetics, The Ohio State UniversityColumbus, OH, USA

**Keywords:** HTLV-1, HTLV-2, p30, p28, accessory proteins

## Abstract

Extensive studies of human T-cell leukemia virus (HTLV)-1 and HTLV-2 over the last three decades have provided detailed knowledge on viral transformation, host–viral interactions and pathogenesis. HTLV-1 is the etiological agent of adult T cell leukemia and multiple neurodegenerative and inflammatory diseases while HTLV-2 disease association remains elusive, with few infected individuals displaying neurodegenerative diseases similar to HTLV-1. The HTLV group of oncoretroviruses has a genome that encodes structural and enzymatic proteins Gag, Pro, and Env, regulatory proteins Tax and Rex, and several accessory proteins from the pX region. Of these proteins, HTLV-1 p30 and HTLV-2 p28 are encoded by the open reading frame II of the pX region. Like most other accessory proteins, p30 and p28 are dispensable for *in vitro* viral replication and transformation but are required for efficient viral replication and persistence *in vivo*. Both p30 and p28 regulate viral gene expression at the post-transcriptional level whereas p30 can also function at the transcriptional level. Recently, several reports have implicated p30 and p28 in multiple cellular processes, which provide novel insight into HTLV spread and survival and ultimately pathogenesis. In this review we summarize and compare what is known about p30 and p28, highlighting their roles in viral replication and viral pathogenesis.

## INTRODUCTION

Human T-cell leukemia virus (HTLV) are complex deltaretroviruses, with HTLV-1 and HTLV-2 causing the most prevalent worldwide infections. HTLV-1 infects approximately 25 million people worldwide and is endemic in Japan, Africa, South America, Iran, and the Caribbean basin (Proietti et al., 2005; Goncalves et al., 2010; Iwanaga et al., 2012). In contrast, HTLV-2 is endemic in Central and West Africa (Goubau et al., 1992; Gessain et al., 1993; Goubau et al., 1993), in native Amerindian populations in North, Central, and South America (Hjelle et al., 1990; Lairmore et al., 1990; Heneine et al., 1991; Levine et al., 1993), and among cohorts of intravenous drug users (IVDUs) in the United States and Europe. Although few HTLV-1 infected individuals (1–5%) develop adult T-cell leukemia/lymphoma (ATL/ATLL) or HTLV-1-associated myelopathy/tropic spastic paraparesis (HAM/TSP), all infected individuals exhibit a persistent antiviral immune response that fails to clear the virus (Poiesz et al., 1980; Yoshida et al., 1982; Takatsuki, 2005; Yoshida, 2005; Lairmore et al., 2012). Furthermore, HTLV-2 infection is not associated with leukemia/lymphoma, although in rare cases it causes a neurodegenerative condition similar to HAM/TSP (Araujo and Hall, 2004).

The genome of HTLV encodes structural and enzymatic genes typical of all retroviruses. In addition, the pX region located between the *env* gene and 3′ long terminal repeat (LTR) encodes four open reading frames (ORF I to IV) with a potential for encoding several proteins (Berneman et al., 1992; Ciminale et al., 1992; Koralnik et al., 1992). As a result of complex splicing, various mRNAs encode regulatory and accessory proteins. Positive regulators of viral gene expression, Tax and Rex, are encoded by a doubly spliced bicistronic mRNA from ORFs IV and III, respectively (Felber et al., 1985; Kiyokawa et al., 1985). Reverse transcription-PCR of mRNA from HTLV-1 infected cell lines and uncultured primary lymphocytes from ATL patients has shown that alternative splicing produces the accessory proteins p12, p30, and p13 (Berneman et al., 1992; Ciminale et al., 1992; Koralnik et al., 1992). A singly spliced mRNA containing ORF-I codes for the accessory protein p12 that can be cleaved to produce a smaller protein, p8 (Van Prooyen et al., 2010). p30 is encoded by a doubly spliced message in which ORF-II is linked to the Tax initiation codon located on exon II, resulting in a 241 amino acid protein. ORF-II also can be singly spliced to produce mRNA that can encode p13 from the internal initiation codon in ORF-II, which corresponds essentially to the last 87 amino acids of p30. Similar studies in the MoT cell line identified accessory proteins in HTLV-2: a bicistronic doubly spliced mRNA encodes p10 and p11 from ORF-I and ORF-V, respectively, and two distinct bicistronic singly spliced mRNAs encoded p28 from ORF II as well as the truncated ORF III isoforms of Rex (Ciminale et al., 1995). p30 and p28 share certain amino acid sequence homology, the last 50 amino acids of p30 share 70% homology with the first 50 amino acids of p28 (Ciminale et al., 1995), and both are nuclear/nucleolar proteins (Koralnik et al., 1993; Ciminale et al., 1995; D’Agostino et al., 1997; Younis et al., 2004). In addition, newly identified proteins, HTLV-1 basic leucine zipper factor (HBZ) and anti-sense protein HTLV-2 (APH-2), are encoded from the antisense genome strand in HTLV-1 and HTLV-2, respectively (Gaudray et al., 2002; Halin et al., 2009).

Tax transactivates viral gene transcription by recruiting transcription factors p300/CREB binding protein (CBP), CREB and AP-1 to the Tax response element (TRE) in the LTR region (Seiki et al., 1986). Tax drives cellular transformation through its ability to alter cellular gene expression, signaling pathways, and cell cycle (Grassmann et al., 2005). Of the factors targeted by Tax, NFκB clearly plays a prominent role in deregulation of cellular gene expression and cellular transformation (Smith and Greene, 1990; Rosin et al., 1998; Robek and Ratner, 1999; Ross et al., 2000). Although Tax is indispensable for viral transformation, the mechanism by which the virus persists *in vivo* leading to T-cell transformation is not clearly understood (Matsuoka and Jeang, 2007). Studies suggest that HBZ and accessory proteins might play a role *in vivo* in HTLV-1 viral persistence and T-cell malignant transformation (Bartoe et al., 2000; Arnold et al., 2006; Arnold et al., 2008; Valeri et al., 2010). Rex binds to the Rex response element (RxRE) on unspliced and singly spliced viral mRNAs to facilitate their nuclear-cytoplasmic export for translation in the cytoplasm (Younis and Green, 2005). p30 and p28 mRNA species can be identified in infected cells (Li and Green, 2007) and in cells from HTLV-1 infected patients (Rende et al., 2011; Bender et al., 2012), albeit at 10^3^–10^4^ lower levels than *tax/rex* mRNA.

Reports identifying antibodies and cytotoxic CD8^+^ T-cells in infected patients with HTLV-1 (symptomatic and asymptomatic) against p30, demonstrate the importance of HTLV-1 accessory proteins in viral persistence and ultimately in the viral life cycle (Jacobson et al., 1992; Chen et al., 1997; Pique et al., 2000). However no studies to date have attempted to identify antibodies or cytotoxic CD8^+^ T-cells against p28 in HTLV-2 infected patients. In this review we will compare the current knowledge on p30 and p28, highlighting the differences and similarities in their roles in the HTLV life cycle.

## *IN VIVO* ROLE OF P30 AND P28

Initial studies to understand the role of p30 were performed by deleting either ORF-I and –II, or ORF II alone from an HTLV-1 infectious molecular clone, which showed that p30 is dispensable for viral gene expression, infectivity, replication, and T-cell immortalization *in vitro* (Derse et al., 1997; Robek et al., 1998). To examine the role of p30 in viral replication and infectivity *in vivo*, T-cell lines immortalized with a viral clone containing mutations ablating both p30 and p13 reading frames (Bartoe et al., 2000) or p30 alone (Silverman et al., 2004) were generated. It was noted that loss of p30 and p13 or p30 alone resulted in a significantly reduced antibody response and lower proviral loads in rabbits indicating that p30 and p13 are required for maintenance of high proviral loads *in vivo* (Bartoe et al., 2000; Silverman et al., 2004). In addition, when p30 alone was ablated, reversion to the wild type sequence was observed, underlining the *in vivo *importance of p30 in maintaining high level viral infection (Silverman et al., 2004). It is noteworthy that these first experiments were performed before the identification of the HTLV-1 HBZ, which is encoded from the anti-sense viral mRNA that overlaps ORFs in the pX region including p30 ORF-II (Gaudray et al., 2002). It is very likely that the mutations ablating p30 expression interfered with the expression of HBZ, which also was shown to be important for viral infectivity *in vivo* (Arnold et al., 2006). Recently, to investigate the relative contribution of HBZ and p30 to viral infectivity *in vivo*, B cell lines expressing viral mutants with specific ablation of HBZ and p30 were generated and used to infect rabbits and macaques. In contrast to the earlier *in vivo* rabbit studies (Silverman et al., 2004), it was shown that viral mutants lacking p30 had little effect on antibody response, infectivity or proviral loads. However, lack of HBZ resulted in reduced proviral loads in rabbits. In addition, there was no evidence of reversion to wild type sequence by the p30 or HBZ mutants (Valeri et al., 2010). Interestingly, in macaque infections it was noted that lack of p30 and HBZ resulted not only in reduced antibody response and infectivity but also reversion to wild type sequence indicating that p30 and HBZ are important for viral infectivity in macaques (Valeri et al., 2010). The discrepancy regarding the requirement for p30 in rabbit infections may be due to the contribution of HBZ to the viral infection. In the same study it was shown that p30 is also required for productive cell-free infection of dendritic cells *in vitro*. The requirement of p30 in terms of *in vitro *viral infection might be cell type-dependent.

On the other hand, an initial report revealed that deletion of the pX region of HTLV-2 causes a reduction in viral replication but not infectivity *in vivo*, but had no effect *in vitro* (Cockerell et al., 1996). To delineate the role of p28, Yamamoto et al. (2008) created a p28 knockout HTLV-2 molecular clone and stably transfected virus producing cells with the wild type HTLV-2 molecular clone or the HTLV-2Δp28 mutant. The irradiated producer cells were co-cultured with human PBMCs, and viral p19 Gag production and cell survival were measured over time. No difference was observed between HTLV-2 and HTLV-2Δp28 indicating that p28 was not required for *in vitro* primary T lymphocyte infectivity, proliferation, and immortalization. The authors also determined the effect of p28 on viral persistence *in vivo* using a rabbit model and found that rabbits infected with HTLV-2 lacking p28 had lower antibody responses and reduced proviral load compared to those infected with wild type HTLV-2 (Yamamoto et al., 2008). The mutation to delete p28 had no effect on the APH-2 gene providing clear evidence that p28 contributes to viral persistence *in vivo*.

Collectively, it is clear that p30 (HTLV-1) and p28 (HTLV-2) are required for efficient viral replication and persistence *in vivo*. Recent findings discussed below provide various possible mechanisms to explain the role of these accessory proteins in viral infection and/or pathogenesis.

## VIRAL GENE EXPRESSION

### TRANSCRIPTIONAL REGULATION

**FIGURE 1:**
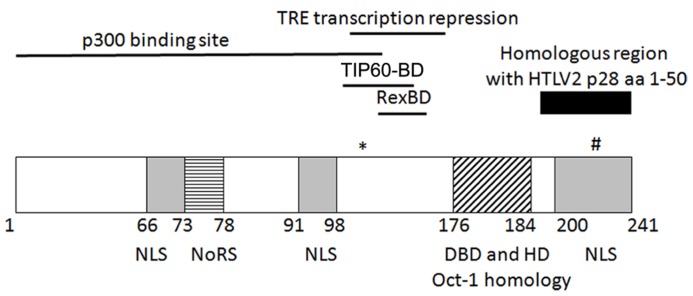
**Schematic representation of the known/putative functional domains of p30.** (*)K106 required for p30 TRE transcriptional repression. (#)T232 required for nucleolus to nuclear transport upon DNA damage. NLS, nuclear localization sequence; NoRS, nucleolar retention sequence; Rex-BD, Rex binding domain; TIP60-BD, TIP60 binding domain; aa, amino acids.

It was demonstrated that p30 is a nuclear/nucleolar localizing protein (Ciminale et al., 1992; Koralnik et al., 1992; see **Figure 1** for summary of key p30 domains). Structurally, p30 contains a bipartite nucleolar/nuclear localization signal (NoRS/NLS), which is comprised of two arginine rich domains (amino acids 73–78 and 91–98; D’Agostino et al., 1997). A later study reconfirmed these regions to be a nucleolar retention signal and also identified two other possible nuclear localization signals in the amino (N) and carboxy (C) termini of p30 (Ghorbel et al., 2006). The latter study also implicated the role of transcription and RNA-interaction in nucleolar/nuclear localization of p30 with the specific interaction of p30 with a 60S ribosomal protein L18a (Ghorbel et al., 2006). Similar to p30, expression of p28 cDNA resulted in nuclear localization of p28 (Ciminale et al., 1995; Younis et al., 2004).

p30 has marked homology with serine and threonine rich transcription factors Oct-1 and-2, Pit-1, and POU-M1 suggesting that p30 could function as a transcriptional regulator (Ciminale et al., 1992). The initial evidence of p30 as a transcriptional regulator was provided by the ability of p30 to differentially modulate CREB responsive promoters by acting as a transactivator when fused to the DNA binding domain of Gal4, and behaving as a repressor when expressed alone. In addition, p30 increased transcription from the TRE at a low concentration but repressed transcription at a higher concentration in the presence and absence of Tax (Zhang et al., 2000). On the other hand, in a similar Gal4 reporter assay, p28 failed to modulate Gal4 DNA binding ability, demonstrating that p28 is devoid of transcriptional activity (Younis et al., 2006). Subsequently, p30 was shown to regulate transcription by binding to CBP/p300. The interaction of p30 and CBP/p300 was evaluated by two hybrid screening, immunoprecipitation, and localization studies. The binding of p30 to p300 was mapped to a highly conserved KIX region of p300, which is also the binding site of Tax. The transcriptional repression of TRE by p30 is a result of competition between p30 and Tax for p300 binding and disruption of the assembly of Tax/CBP/P300 on the TRE (Zhang et al., 2001). The p300 binding region was mapped to amino acids 1–132 whereas the TRE transcription repression function of p30 was mapped to amino acids 100–179. This 80 bp region contains five of the six lysine residues in p30 of which lysine 106 was found to be important for repression. Unlike wild type p300, mutant p300 with impaired histone acetyltransferase activity (HAT) was unable to rescue p30-mediated LTR transcription repression. Moreover, p30 LTR repression was enhanced by histone deacetylase (HDAC-1) and inhibited by Trichostatin A (inhibitor of HDAC-1). These data suggest that the LTR transcription repression function of p30 could be regulated by acetylation on lysine or that these effects are due to global effects of HATs and HDACs on transcription (Michael et al., 2006).

p30 has the ability to transcriptionally activate numerous cellular genes. In one study it was shown that p30 binds and stabilizes the Myc/Tat-interacting protein (TIP60) transcriptional complex (Awasthi et al., 2005). Furthermore, p30 binds and recruits the 60-kDa (TIP60) to the Myc transcription complex to activate Myc responsive genes, which requires the HAT of TIP60. In addition, the transcriptional activity of p30 was dependent on transforming transcriptional activator protein (TRRAP)/p434. The idea that p30 plays a role in transformation by enhancing Myc-responsive genes was supported by the data showing that p30 cooperated with Myc in focus-forming transformation assays (Awasthi et al., 2005). More evidence for regulation of cellular transcription by p30 comes from a study showing that p30 can interact with the PU.1 transcription factor through its DNA binding domain resulting in the suppression of PU.1 DNA binding. Subsequently, the expression of p30 decreased cell surface expression of TLR4 as the expression of TLR4 is directly under the influence of PU.1 transcription activation. Concurrently, reduced TLR4 expression by p30 resulted in reduced pro-inflammatory cytokines TNF-α, MCP-1 and IL-8 whereas the expression of anti-inflammatory cytokine IL-10 was increased. Additionally it was found that p30 increased phosphorylation of GSK3-β leading to an increase in IL-10 production (Datta et al., 2006). These data indicate that p30 can influence innate and adaptive immunity to potentially facilitate viral spread by influencing cellular transcription. Similar experiments to investigate the ability of p28 to regulate transcription of cellular genes demonstrated that unlike p30, p28 has no transcription function, although chromatin immunoprecipitation assays (ChIP) show that p28 is recruited to the LTR along with the transcriptional machinery (Younis et al., 2006).

In order to further characterize the role of p30 in cellular transcription, various groups have performed microarray analysis. The initial microarray analysis showed that p30 altered expression of cellular genes involved in apoptosis, cell cycle regulation, cell adhesion, transcription, and T-cell signaling and activation. It was found that p30 selectively activates genes involved in T cell signaling/activation and also enhances transcription mediated by NFAT, NF-κB, and AP-1 when stimulated (Michael et al., 2004). The ability of p30 to transcriptionally activate numerous cellular genes was also reported, where p30 binds and stabilizes the Myc/TIP60 transcriptional complex (Awasthi et al., 2005). Recently, similar microarray analysis also showed that p30 was capable of altering transcription of cellular genes involved in apoptosis and transcription (Taylor et al., 2009). Overall, these data indicate that p30 regulates transcription of viral genes as well as cellular genes to create an environment that promotes viral gene expression and survival of infected cells.

### POST-TRANSCRIPTIONAL REGULATION

HTLV-1 p30 controls viral gene expression at the post-transcriptional level by interacting with and retaining *tax/rex* mRNA in the nucleus. The expression of p30 results in decreased levels of *tax/rex* mRNA specifically: this effect was not observed with other viral mRNAs. p30 binds to the second splice junction of *tax/rex* mRNA, which then is retained in the nucleus to repress tax expression and, in turn, viral gene expression (Nicot et al., 2004). HTLV-2 p28 mediates nuclear retention of the tax/rex mRNA via a similar post-transcriptional mechanism (Younis et al., 2004). Younis et al. (2006) went on to demonstrate that both p28 and p30 are recruited by Tax to the viral promoter, which then bind to their respective *de novo* transcribed response elements leading to the retention of *tax/rex* mRNA in the nucleus.

Rex and p30 have antagonistic functions where the Rex protein facilitates the nuclear-cytoplasmic export of unspliced and singly spliced mRNA while p30 retains *tax/rex* mRNA in the nucleus. The Rex and p30 interaction is favored when p30 is bound to *tax/rex* mRNA. The interaction of p30 with Rex has no effect on the ability of Rex to export mRNA from the nucleus to the cytoplasm whereas Rex moderately rescues the nuclear retention of *tax/rex* mRNA in the nucleus by p30. This interplay between p30 and Rex may be critical to establishing HTLV-1 latency (Sinha-Datta et al., 2007). Immunofluorescence confocal microscopy showed that p30 and Rex localized with CRM1, suggesting an interaction between Rex and p30 in HTLV-1 post-transcriptional control (Baydoun et al., 2007). On the other hand, p28 inhibited *tax/rex* mRNA export through the TAP/p15 pathway and not CRM1, where exogenous expression of TAP/p15 rescued p28 post-transcriptional repression (Younis et al., 2006).

Recently, microarray studies demonstrated that p30 can regulate cellular gene expression at the post-transcriptional level as well. Cytoplasmic expression of genes involved in cell signaling, transcription, translation, replication, cytoskeleton, DNA, and metabolism was decreased while those involved in repair, apoptosis, cell adhesion, and signaling were increased (Taylor et al., 2009). Studies to evaluate the effect of p28 post-transcriptional regulation on cellular genes have not been performed and are worth investigating.

## CELL CYCLE REGULATION

Cell cycle control is an intricate, highly regulated pathway that guarantees proper cellular turnover and involves various cyclin dependent kinases (CDK) and Cyclins that phosphorylate key molecules allowing cell cycle progression. Different studies have shown that p30 also influences cell cycle progression. The G2/M checkpoint is regulated by a tight balance of phosphorylation and dephosphorylation events, where Cdc25c plays a crucial role in the onset of mitosis (Draetta and Eckstein, 1997). p30 activates the G2/M checkpoint by increasing the phosphorylation of Chk1 and subsequently increasing phosphorylation of Cdc25c. Another mechanism of p30-induced G2/M cell cycle arrest would be via reduction of PLK1 levels and phosphorylation resulting in decreased phosphorylation of Cdc25. In support of this mechanism, cells immortalized with an HTLV-1 molecular clone lacking p30 were more susceptible to apoptosis when treated with camptothecin and etoposide (Datta et al., 2007).

In addition to the effects of p30 on the G2M checkpoint, p30 activates Myc-driven genes leading to increased S phase progression and polyploidy (Awasthi et al., 2005). However, a recent study reported that expression of p30 delays S phase entry (Baydoun et al., 2010). The CDK2–Cyclin E complex is known to phosphorylate Rb, which in turn releases the transcriptional factor E2F, thereby controlling the expression of several genes required for S phase entry. It was shown that p30 delays S phase entry by interacting with both Cyclin E and CDK2 affecting their complex formation and resulting in reduced phosphorylation of Rb (Vermeulen et al., 2003). Hence, p30 expression causes a decrease in E2F and Cyclin E levels, and an increase in p21^Waf^ expression. In contrast to p30, the expression of p28 had no effect on S phase entry and p28 was incapable of binding to Cyclin E (Baydoun et al., 2010).

## DNA DAMAGE AND REPAIR

The integrity of the genome is essential for cell propagation, and timely repair of DNA damage is equally important to avoid the accumulation of mutations that could result in tumorigenesis. Retrovirus integration elicits the double stranded DNA damage response where cells must repair DNA damage in order to avoid apoptosis. DNA double strand breaks (DSB) are highly toxic to cells and are associated with developmental, immunological, neurological disorders, and various cancers (Jackson and Bartek, 2009; McKinnon, 2009). Anupam et al. (2011) demonstrated that p30 confers a growth advantage to cells that have undergone double-stranded DNA damage. It was shown that p30 interacts with ATM and reduces the levels of ataxia telangiectasia mutated (ATM) and phosphorylated ATM upon double stranded DNA damage. Mass spectrometric studies identified REGγ, a nuclear proteasome activator, as a cellular binding factor of p30 that might target ATM for degradation. This mechanism of p30 targeting ATM to the proteasome for degradation through interaction with REGγ is consistent with co-elution of ATM, p30, and REGγ in the same size exclusion chromatography fractions (Anupam et al., 2011). Lowering the levels of ATM by p30 upon DNA damage would decrease p53-mediated apoptosis thereby facilitating survival of infected cells. Interestingly, the levels of p30 corresponded to the levels of REGγ (Anupam et al., 2011). Another study showed that p30 translocates from the nucleolus to the nucleus upon DNA damage via phosphorylation at threonine 232, which is part of a mitogen-activated protein kinase (MAPK) domain in the carboxy terminus. The expression of p30 inhibits homologous recombination and favors non-homologous recombination in natural and induced DNA damage conditions. p30 inhibits homologous DNA damage by interfering with the assembly of the MRE11/RAD50/NBS1 (MRN) complex by interacting with RAD50 and NBS1. The amino terminal of p28 has a similar MAPK domain except for a proline in the place of threonine. Mutating this proline to threonine to mimic the MAPK domain of p30 did not enable p28 to alter DNA repair (Baydoun et al., 2011). The interaction between p30 and REGγ was confirmed by another study using a similar mass spectrometric technique (Ko et al., 2013). However, this study showed that the ability of p30 to retain *tax/rex* mRNA in the nucleus was dependent on recruiting REGγ to the *tax/rex* mRNA by p30. Further, reduced levels of REGγ resulted in increased HTLV-1 gene expression (Ko et al., 2013). In contrast, another study showed that reduced levels of REGγ had no effect on HTLV-1 gene expression (Doueiri et al., 2012). The discrepancy about the role of p30 and REGγ binding could be explained by the use of different cell lines and techniques.

## p30 AND p28 INTERACTOMES

The comparative studies of HTLV-1 and HTLV-2 are useful to highlight the differences that might be responsible for HTLV-1 pathogenesis. Studies investigating host–viral protein interactions have been centered on Tax-1 and Tax-2. Moreover, a large amount of work has focused on p30 because HTLV-1 is pathogenic while similar comparative studies have not been performed on p28. Our group performed a mass spectrometry-based analysis to compare the p30 and p28 interactomes in terms of host–protein interactions. The mass spectrometry results suggested that p30 interacts with a larger and wider range of proteins mainly involved in cell cycle regulation, cell survival, DNA repair, cancer pathways, protein post-translational modifications, and metabolism, whereas p28 interacts with a smaller number of proteins that are involved in mRNA processing and protein post-translational modifications. These data are consistent with the known functions of p30 and p28. Furthermore, we confirmed the interaction between p30 and REGγ and also showed that p28 does not interact with REGγ. Tagging RNA for export is a complex process that involves several proteins involved in splicing such as the SR protein complex and hnRNPs (Caputi and Zahler, 2002; Han et al., 2010; Busch and Hertel, 2012). The exact mechanism utilized by p28 and p30 to retain *tax/rex* mRNA is not known; however, the mass spectrometry analyses identified interactions between p28 and hnRNP H1 and hnRNP F. Conversely, p30 does not have an interaction with hnRNP H1(Doueiri et al., 2012). However, hnRNP K was identified to interact with both p30 and p28. We postulate that p30 and p28 might use these interactions to modulate the role of hnRNPs or other proteins involved in RNA export, which could be a possible mechanism for retention of *tax/rex* mRNA in the nucleus. However the full implication of these interactions warrants further investigation. In addition, a novel interaction between p30 and NEAP interacting protein 30 (NIP30) was also identified. NIP30 is predicted to bind DNA binding/EF hand/Leucine zipper protein (NEFA), which has been shown to localize to the Golgi complex. Preliminary characterization of NIP30 indicated that it is a nuclear protein (Simpson et al., 2000). Since the biological function of NIP30 is not known, further investigation is required to understand the importance of the interaction between p30 and NIP30 (Doueiri et al., 2012).

**FIGURE 2:**
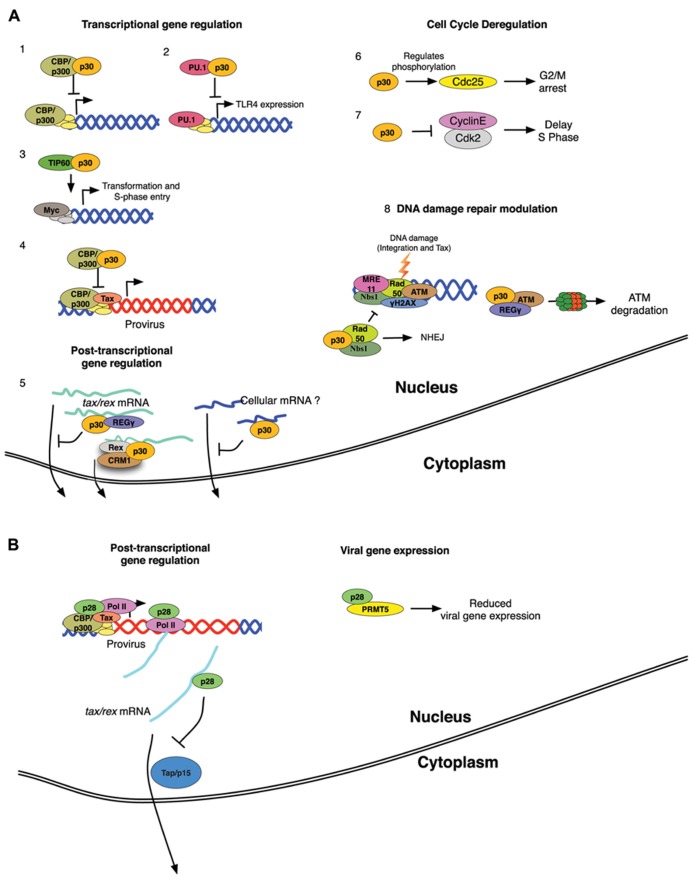
**(A)** Schematic representation of p30 functions. (1) Interaction of p300 and p30 influences cellular gene expression (2) p30 inhibits PU.1 transcriptional activity, affecting TLR4 expression and ultimately the innate immune response. (3) p30 activates Myc-mediated transcription by recruiting TIP60. (4) p30 disrupts p300 recruitment to the TRE by Tax thereby reducing viral gene transcription. (5) p30 inhibits tax/rex mRNA export possibly through REGγ interaction, Rex counteracts p30 inhibitory function. (6) p30 causes increased phosphorylation in cdc25C on S216 and reduced phosphorylation on S198 leading to G2/M checkpoint activation. (7) p30 disrupts Cyclin E-CDK2 complex formation causing delayed S phase entry. (8) p30 interaction with Regγ down-regulates ATM levels, in addition p30 inhibits MRN complex formation during DNA damage activating NHEJ. **(B) **Schematic representation of p28 functions. p28 is recruited by Tax to the viral promoter where it co-migrates with the transcriptional machinery until the response element is made. p28 interacts with tax/rex mRNA and inhibits its nuclear export by modulating the Tap/p15 pathway. p28 interaction with PRMT5 leads to reduced viral gene expression.

Interestingly, both p30 and p28 interact with protein arginine methyltransferase 5 (PRMT5). It has been shown that PRMT5 can regulate transcription and also is involved in mRNA processing, mainly splicing (Karkhanis et al., 2011). Moreover, PRMT5 is up-regulated in B cell lymphomas and most transformed cell lines (Wang et al., 2008). The significance of the PRMT5 interaction was investigated by evaluating the effect of PRMT5 knockdown on viral gene expression. Intriguingly, lower levels of PRMT5 significantly reduced HTLV-2 gene expression, whereas no such effect was seen on HTLV-1 gene expression. It remains to be determined whether the p28–PRMT5 interaction regulates HTLV-2 gene expression at the transcriptional or post-transcriptional level. The interaction between p30 and PRMT5 could be important at a stage of viral spread and/or pathogenesis other than gene expression. This study provides a novel hypothesis about the role of p30 and p28 in the different pathological outcomes of HTLV-1 and HTLV-2 (Doueiri et al., 2012). A summary of various molecular process in which HTLV-1 p30 and HTLV-2 p28 are involved is shown in **Figure 2**.

## SUMMARY

Human T-cell leukemia virus (HTLV)-1 and HTLV-2 are related retroviruses that have different pathological outcomes. While HTLV-1 infection is linked to cancer, inflammatory and neurodegenerative diseases, HTLV-2 is minimally pathogenic. The differences in the viral oncoprotein Tax-1 and Tax-2 are in part responsible for the distinct outcomes of infection with these viruses. However the absence of Tax expression in most ATL patients and the requirement for the accessory genes in HTLV persistence in *in vivo* models demonstrate a more complex viral regulatory mechanism that allows the virus to infect and persist in the host. HTLVs encode various proteins that affect multiple signaling nodes (Simonis et al., 2012) thereby ensuring tight control of the cellular mechanisms required for infectivity, persistence, and spread, while down-regulating those involved in limiting viral spread. HTLV-1 p30 and its homologue HTLV-2 p28 are accessory proteins required for viral persistence *in vivo*, but are dispensable for *in vitro* viral persistence. p30 and p28 are both post-transcriptional negative regulators of viral replication; they interact with *tax/rex* mRNA and retain it in the nucleus, thereby modulating oncogenic/immunogenic Tax expression allowing the virus to escape immune surveillance while slowly transforming the infected cells. In addition, p30 functions in transcriptional and post-transcriptional mechanisms independent of Tax. p30 causes G2/M cell cycle arrest and delays S phase entry, down-regulates ATM and favors NHEJ, modulates the innate immune response and increases the expression of genes involved in T-cell survival and expansion, while down-regulating genes involved in apoptosis. Interestingly, although the first 50 amino acids of p28 share 77% homology with the last 50 amino acids of p30, this polypeptide domain is devoid of transcriptional activity or effects on the host cell cycle, DNA damage response or immune response. Proteome analysis revealed that p30 and p28 have distinct interactome profiles even though they both interact with some similar proteins; therefore, p30 and p28 must have divergent functions during the lifecycle of the viruses.

Comparison of the roles of p30 and p28 has provided further insight into HTLV biology. Further studies are needed to determine their distinct roles in viral latency, low penetrance and ultimately the differential pathological outcomes of HTLV-1 and HTLV-2.

## Conflict of Interest Statement

The authors declare that the research was conducted in the absence of any commercial or financial relationships that could be construed as a potential conflict of interest.
